# A Multimodal Fuzzy Approach in Evaluating Pediatric Chronic Kidney Disease Using Kidney Biomarkers

**DOI:** 10.3390/diagnostics14151648

**Published:** 2024-07-30

**Authors:** Cristian Petru Dușa, Valentin Bejan, Marius Pislaru, Iuliana Magdalena Starcea, Ionela Lacramioara Serban

**Affiliations:** 1 Department of Pediatrics, Faculty of Medicine, “Grigore T. Popa” University of Medicine and Pharmacy, 700115 Iasi, Romania; 2 Department of Surgery, Faculty of Medicine, “Grigore T. Popa” University of Medicine and Pharmacy, 700115 Iasi, Romania; 3 Department of Engineering and Management, Faculty of Industrial Design and Business Management, “Gheorghe Asachi” Technical University of Iași, 700050 Iasi, Romania; 4 Department of Morpho-Functional Sciences II, Discipline of Physiology, “Grigore T. Popa” University of Medicine and Pharmacy, 700115 Iasi, Romania

**Keywords:** pediatric, chronic kidney disease, fuzzy logic, neutrophil gelatinase-associated lipocalin, system modeling, diagnostic support

## Abstract

Chronic kidney disease (CKD) is one of the most important causes of chronic pediatric morbidity and mortality and places an important burden on the medical system. Current diagnosis and progression monitoring techniques have numerous sensitivity and specificity limitations. New biomarkers for monitoring CKD progression have been assessed. Neutrophil gelatinase-associated lipocalin (NGAL) has had some promising results in adults, but in pediatric patients, due to the small number of patients included in the studies, cutoff values are not agreed upon. The small sample size also makes the statistical approach limited. The aim of our study was to develop a fuzzy logic approach to assess the probability of pediatric CKD progression using both NGAL (urinary and plasmatic) and routine blood test parameters (creatinine and erythrocyte sedimentation rate) as input data. In our study, we describe in detail how to configure a fuzzy model that can simulate the correlations between the input variables ESR, NGAL-P, NGAL-U, creatinine, and the output variable Prob regarding the prognosis of the patient’s evolution. The results of the simulations on the model, i.e., the correlations between the input and output variables (3D graphic presentations) are explained in detail. We propose this model as a tool for physicians which will allow them to improve diagnosis, follow-up, and interventional decisions relative to the CKD stage. We believe this innovative approach can be a great tool for the clinician and validates the feasibility of using a fuzzy logic approach in interpreting NGAL biomarker results for CKD progression.

## 1. Introduction

The emergence of cases of chronic kidney disease (CKD) in patients of pediatric age has been increasing over the past 10 years. Diagnosing and monitoring these patients remains a current challenge, as there is an estimate that almost 1% of the total pediatric population may present one of the five different stages of CKD [1]. The treatment of these patients is also a demanding challenge, but there is evidence that intervening at an early stage of CKD can slow the progression of the disease [2]. In the last 50 years, in the diagnosis and monitoring of these patients, the gold standard is represented by blood and urine tests with sensitivity and specificity that can be improved, such as the evaluation of creatinine clearance (either laboriously with inulin, or indirectly with formulas), inflammatory syndrome, albuminuria, and proteinuria, markers that are not intrinsic to the kidneys [3,4]. Creatinine is a muscle metabolite with elimination in the kidney. Its values can be changed by muscle mass, external intake, and the degree of physical exertion performed. The assessment of renal function is indirect and will therefore vary according to these factors [3]. Due to the aforementioned limitations, it was suggested to find new or complementary biological methods superior to the use of creatinine clearance. In this regard, new molecules were sought to allow better diagnosis and monitoring of the patient, such as NGAL (neutrophil gelatinase-associated lipocalin), KIM-1 (kidney injury molecule 1), cystatin-C, IL-18, L-FABP (liver-type fatty-acid binding protein), N-Acetyl-β-D-glucosaminidase or TIMP-2 and IGFBP-7 [5,6,7,8]. Of those listed above, NGAL exhibits many qualities [9,10] These biomarkers have been studied extensively in adult populations [11,12,13]. While the results are encouraging, pediatric studies in this area are few and insufficient [14,15,16]. Most pediatric studies have small patient numbers, which makes a statistical approach biased [16,17,18,19]. Furthermore, there is no consensus regarding the NGAL pathological value range [16,17,18,19]. There is a need for a different approach to evaluating the impact and utility of these biomarkers in the CKD pediatric studies. Fuzzy logic mimics the medical diagnostic process. It allows for suboptimality and uncertainty, while providing a reliable soft computing technique of data interpretation [20,21,22]. In this study, we propose an innovative multimodal fuzzy approach to evaluating pediatric chronic kidney disease using NGAL (both plasmatic and urinary) as kidney biomarkers. 

## 2. Materials and Methods

From a methodological point of view, the proposed approach is fuzzy logic. There are several key points that highlight the strengths and advantages of fuzzy logic in dealing with the complexities and uncertainties inherent in medical diagnostics:

*Handling Uncertainty and Vagueness*. Medical diagnosis often involves subjective assessments and imprecise information. Symptoms or dizziness are inherently vague and can vary in intensity and description from patient to patient. Unlike traditional binary logic systems, which require precise inputs, fuzzy logic systems can work with degrees of truth. For instance, a symptom like “mild fever” can be represented as a fuzzy variable with a value between “low” and “high”, rather than a binary state of “fever” or “no fever”.

*Modeling Complex Relationships.* Medical conditions are often influenced by a complex interplay of various symptoms, patient history, genetic factors, and environmental influences. Fuzzy logic can model these complex relationships through the use of fuzzy rules. These rules can incorporate multiple variables and their interdependencies. For example, a fuzzy rule might state: “IF the patient has a mild fever AND moderate headache AND slight nausea, THEN there is a possibility of condition X”. This approach mirrors the reasoning process of medical professionals more closely than strict binary logic.

*Integration of Multidimensional Data*. Diagnostic processes often require the integration of data from various sources, including physical symptoms, lab results, and patient history. Fuzzy logic systems can handle and integrate these multidimensional data seamlessly. They allow for the combination of qualitative and quantitative data, providing a more holistic diagnostic assessment. 

A study published in 2022 reveals that between 2000 and 2021 there were 479 publications on the topic of fuzzy logic in medicine. Medline was used to identify the medical publications with fuzzy logic [23].

**Figure 1:**
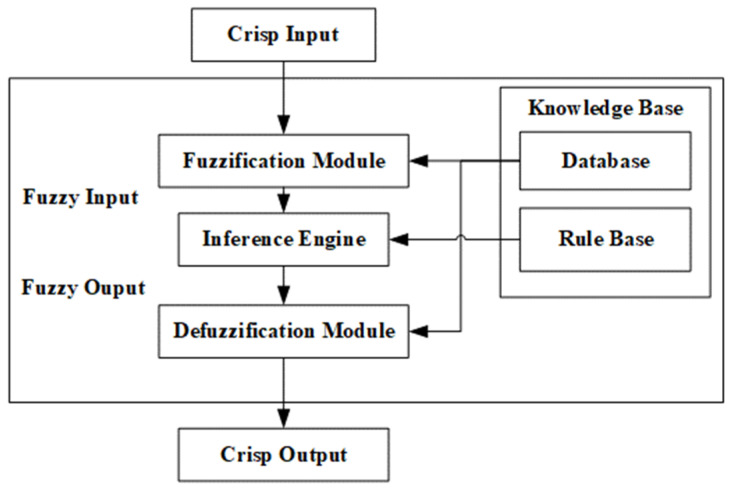
The main components of a fuzzy logic system (FLS).

In principle a fuzzy logic system (FLS) consists of several key components that work together to process inputs and produce outputs based on fuzzy set theory. The main components of a fuzzy logic system are shown in Figure 1. The *Fuzzification Module* converts crisp inputs to fuzzy values using membership functions. The *Knowledge Base* stores fuzzy rules and membership functions. The *Inference Engine* applies fuzzy rules to fuzzified inputs to generate fuzzy outputs. The *Defuzzification Module* converts fuzzy outputs back to crisp values.

Concerning the Knowledge Base, ours used both current literature values and our own prospective study, performed using data gathered both from the observation charts and biological samples (blood and urine) of patients admitted during June 2021–August 2023, in the Nephrology department of Sf. Maria Hospital of Iași, Romania. For our study, blood and urine samples (5 mL each) were obtained, centrifuged and supernatant was redistributed into a minimum of 1 mL/Eppendorf tube. The tubes were initially chilled at −20 °C degrees, and later to −80 degrees Celsius. Once all patients were gathered, probes were brought to room temperature and both serum and urinary NGAL were tested using the *Human Neutrophil Gelatinase-Associated Lipocalin/NGAL (LCN2) ELISA Kit* from producer abbexa, according to the manufacturer’s instructions. The data were anonymized and confidential. 

The study was conducted in accordance with the Declaration of Helsinki, and the protocol was approved by both the Ethics Committees of the Sf. Maria Hospital (no. 5075/17 February 2021) and of the University of Medicine and Pharmacy “Grigore T. Popa” Iași (no 8753/28 May 2020).

**Figure 2:**
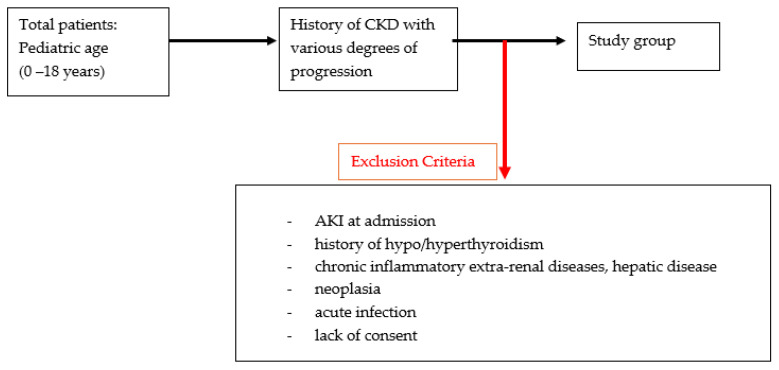
Flow-chart regarding the inclusion and exclusion criteria of the patients.

Rigorous inclusion and exclusion criteria were applied (Figure 2). Included patients were required to be of pediatric age (0–18 years) and history of diagnosis of chronic kidney disease (according to the KDIGO definition criteria). Exclusion criteria included AKI at admission (so as not to create confounding variation of NGAL values), history of hypo/hyperthyroidism, chronic inflammatory extra-renal diseases, hepatic disease or neoplasia, and acute infection (all of which can induce increases in NGAL) and lack of consent to be included in the study.

Between June 2021 and August 2023, 17 patients who met the aforementioned criteria were enrolled. 

Of the 17, only 16 agreed to provide a urinary sample. All agreed to give blood samples. The data collected included 38 different parameters. Some of the parameter distribution is represented in Table 1. Out of these, only 4 were used in the study: erythrocyte sedimentation rate (ESR), serum creatinine (creat), urinary NGAL (NGAL-U), and plasma NGAL (NGAL-P).

Our patients presented multiple etiologies for chronic kidney disease including atypic hemolytic uremic syndrome, corticoresistant nephrotic syndrome, corticodependent nephrotic syndrome, vesicoureteral reflux (degrees 2 to 4) with signs of reflux nephropathy, crescent glomerulonephritis with Ig A deposits, and neurogenic bladder with reflux nephropathy. There were no stage 2 CKD patients; other stages were represented.

Patient characteristics regarding the fuzzy logic parameters and associated derived rules for our FIS model are presented in Table 2. These values were used for modeling and verification. 

Progression of CKD was defined as a persistent creatinine clearance (eGFR calculated by Schwartz formula) decrease of >4 mL/min/1.73 m^2^ over the last year. Gray was considered as persistent creatinine clearance decrease between 1 and 4 mL/min/1.73 m^2^. No progression was considered as less than 1 mL/min/1.73 m^2^.

The main values of plasma/serum NGAL and urinary NGAL reported in the current literature in relationship to CKD progression can be viewed in Table 3. 

Summing up the literature data, we considered that regarding CKD, there are propositions of pathological implications from values of NGAL-P of 50–1405 ng/mL. We also consider that below 10 ng/mL, there is no pathological implication. Between 10 and 200, there is a gray area that needs more validation. 

For NGAL-U, the literature suggests pathological implications from values of 14 ng/mL to 734 ng/mL. We consider that below 6 ng/mL there should be no pathological implication. However, we propose a gray area between 10 and 100 ng/mL that needs further validation. 

ESR has a theoretical normal value range from 0 to 11 mm/1 h. However, our experience is that starting from an ESR of 6 and above, we can observe the presence of CKD related inflammation and that 5–8 is a gray area. This observation is based on our patient sample size.

Finally, regarding creatinine, while we consider the range of 0 to 1.2 mg/dL as normal, we also propose a gray area of interpretation between 0.2 and 0.8 that will mitigate the relationship with age, weight, height, and muscle mass. To further account for this in our modeling, we propose our pathological interval should arise from 0.6. The overlap of these membership functions illustrates the variation of interpretation of creatinine values in clinical practice. 

Creating a fuzzy model involves several stages, as follows: 

*Problem definition and System:* Define the problem or process to be modeled, including the input and output variables.

*Fuzzification:* Convert the crisp input values into fuzzy values using membership functions. Define the fuzzy sets and corresponding membership functions for each input variable.

*Rule Base Creation:* Develop a set of fuzzy rules that describe the system’s behavior. These rules are typically in the form of “IF–THEN” statements, combining fuzzy variables.

*Inference Mechanism:* Apply the fuzzy rules to the fuzzified inputs to obtain fuzzy outputs. Use inference methods such as those of Mamdani or Sugeno to process the rules.

*Defuzzification:* Convert the fuzzy output back into a crisp value. Select a defuzzification method (e.g., centroid, bisector, or mean of maxima) to achieve this.

The configuration of the model is performed in the MATLAB R2023b environment, using the Fuzzy System Designer application.

The configuration steps are presented below.

### 2.1. Problem Definition and System

Our objective is to develop a fuzzy logic approach to assess the probability of pediatric CKD progression using both NGAL (urinary and plasmatic) and routine blood test parameters (creatinine and ESR) as input data.

**Figure 3:**
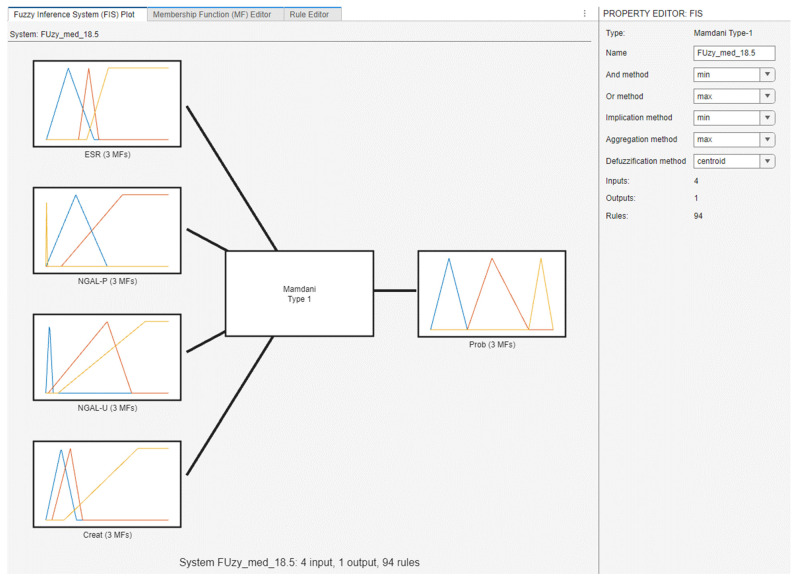
Fuzzy inference system (FIS).

For the fuzzy model, we have taken into consideration four biological parameters: the erythrocyte sedimentation rate (ESR), serum creatinine (Creat), urinary NGAL (NGAL-U), and plasma NGAL (NGAL-P). The reasoning behind this choice of parameters was that, considering chronic kidney disease is a multifactorial condition, each one represents and evaluates the different etio-pathogenic systems and subsystems involved. Consequently, the four-input one-output problem must be discussed and solved in order to determine the probability of CKD (Figure 3).

Figure 3 shows a screenshot of the fuzzy inference system (FIS) in MATLAB’s Fuzzy Logic Designer. The fuzzy inference system has four input variables (ESR, NGAL-P, NGAL-U, Creat) and one output variable (Prob), with each variable having three membership functions. The type of fuzzy inference system used here is Mamdani Type-1. The defuzzification method is set to centroid (as can be seen in Figure 3) which calculates the center of the area under the curve of the aggregated output membership functions.

### 2.2. Fuzzification

The first step is to collect the crisp inputs, *ESR*, *Creat*, *NGAL-P*, and *NGAL-U,* and to assess the degree to which these inputs belong to the appropriate fuzzy sets. A crisp input is a numerical value limited to the universe of discourse. The ranges of the universe of discourses were determined by our datasets’ values. Fuzzy sets can have a variety of shapes, as they model the value distribution curves.

**Figure 4:**
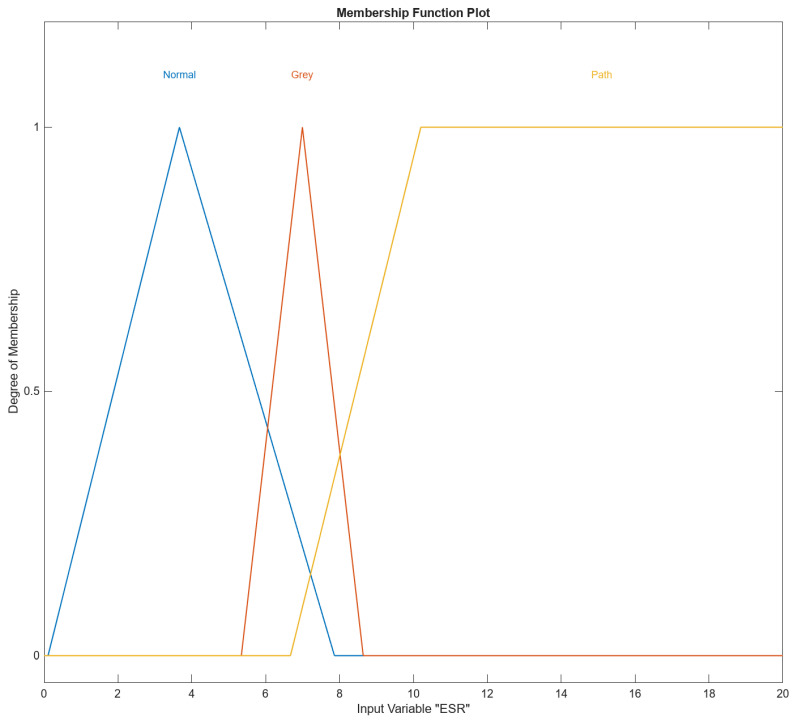
The ESR input variable with its three membership functions.

**Figure 5:**
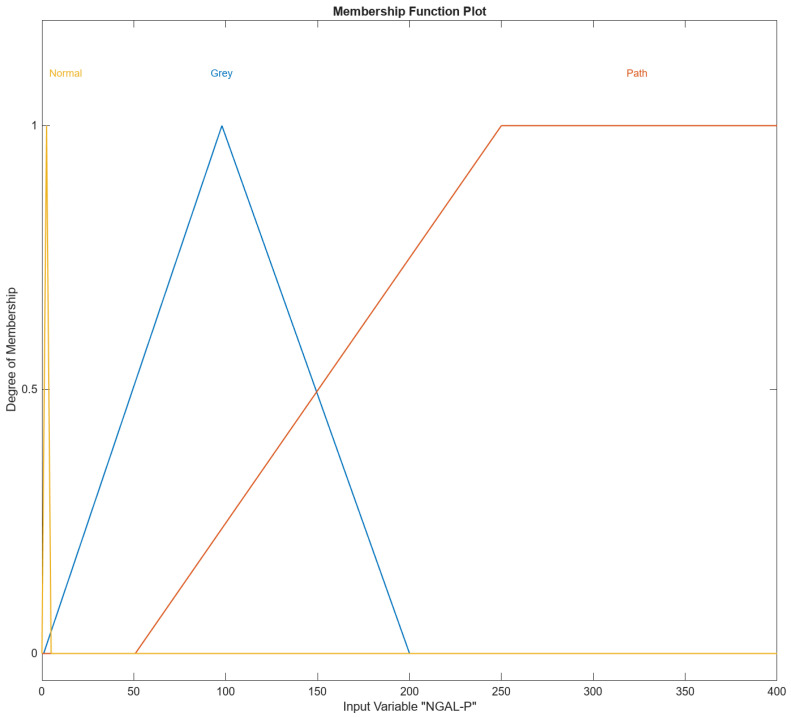
The NGAL-P input variable with its three membership functions.

**Figure 6:**
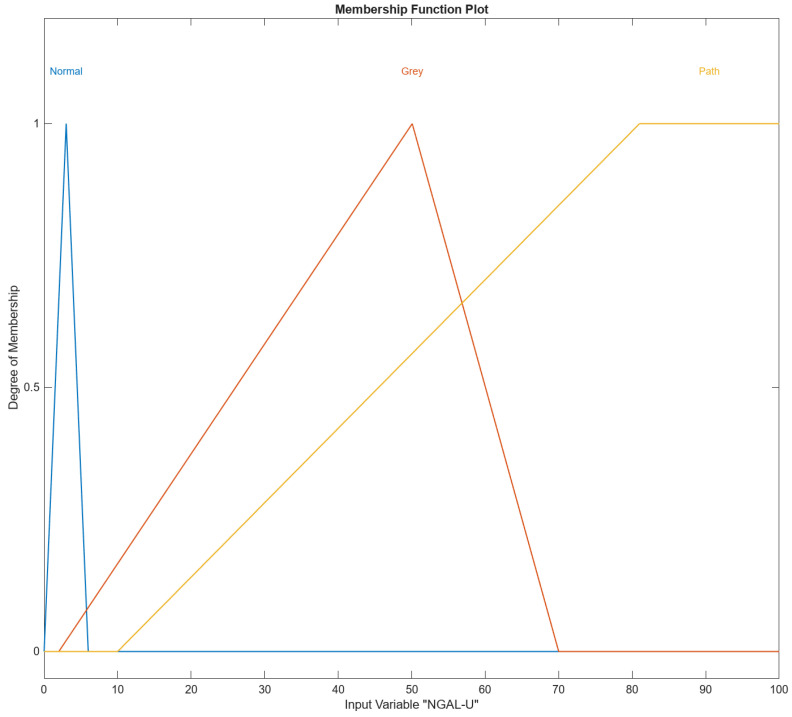
The NGAL-U input variable with its three membership functions.

**Figure 7:**
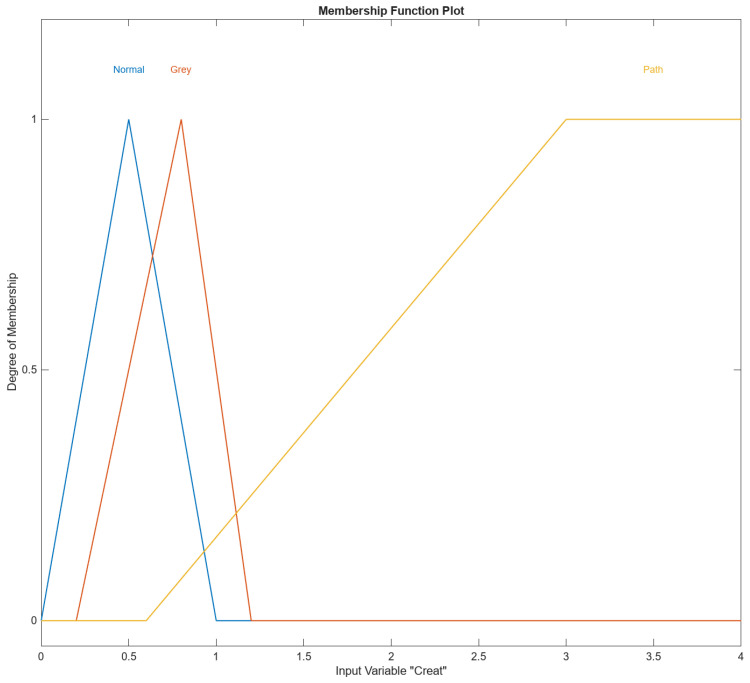
The Creat input variable with its three membership functions.

**Figure 8:**
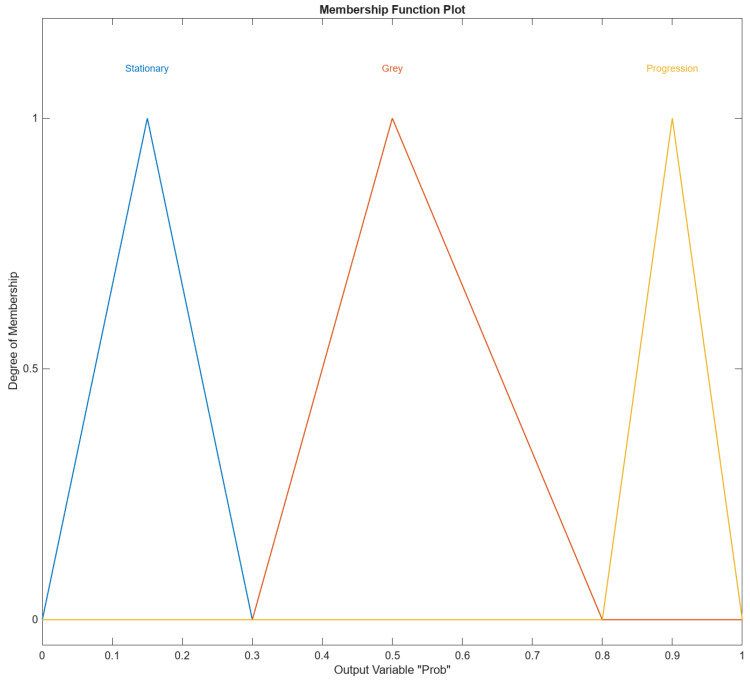
The Prob output variable with its three membership functions.

In Figure 4, Figure 5, Figure 6, Figure 7 and Figure 8, the membership functions used as the linguistic variables are presented. For all the input parameters, to respect scientific realities, and at the same time for a smoother response of modeled fuzzy system, an adequate overlap in adjacent fuzzy sets is present.

Figure 4 shows the Membership Function (MF) for the input variable ESR in MATLAB R2003b’s Fuzzy Logic Designer. The range of the input variable is from 0 to 20. Names of the membership functions are “Normal”, “Gray”, and “Path”. ESR “Normal” membership function is triangular and is defined by the parameters [0.105, 3.6623, 7.86]. This triangular membership function peaks at approximately 3.66 and tapers off to zero around 0.105 and 7.86 and represents the “Normal” range of the input variable ESR. The ESR “Gray” membership function is triangular and is defined by the parameters [5.33916, 6.99316, and 8.63916]. This triangular membership function peaks at approximately 6.99 and tapers off to zero around 5.34 and 8.64 and represents the “Gray” range of the input variable ESR. The ESR “Path” membership function is trapezoidal and is defined by the parameters [6.67, 10.2, 20, and 21]. This trapezoidal membership function starts rising at 6.67, reaches full membership at 10.2, and maintains full membership up to 20, tapering off to 0 at 21, and represents the “Path” range of the input variable ESR.

Figure 5 shows the Membership Function (MF) the input variable NGAL-P in MATLAB’s Fuzzy Logic Designer. The range of the input variable is from 0 to 400. Names of the membership functions are “Normal”, “Gray”, “Path”. NGAL-P “Normal” membership function is triangular and is defined by the parameters [0, 2.5, 5]. This triangular membership function peaks at 2.5 and tapers off to zero at 0 and 5 and represents the “Normal” range of the input variable NGAL-P. NGAL-P “Gray” membership function is triangular and is defined by the parameters [0.923, 97.9183, 200]. This triangular membership function peaks at approximately 97.9183 and tapers off to 0 around 0.923 and 200 and represents the “Gray” range of the input variable NGAL-P. The NGAL-P “Path” membership function is trapezoidal and is defined by the parameters [50.8, 249.961, 402, 415]. This trapezoidal membership function starts rising at 50.8, reaches full membership at 249.961, maintains full membership up to 402, and tapers off to 0 at 415 and represents the “Path” range of the input variable NGAL-P.

Figure 6 shows the membership function (MF) the input variable NGAL-U in MATLAB’s Fuzzy Logic Designer. The range of the input variable is from 0 to 100. The names of the membership functions are “Normal”, “Gray”, and “Path”. The NGAL-U “Normal” membership function is triangular and is defined by the parameters [0, 3, and 6]. This triangular membership function peaks at 3 and tapers off to zero at 0 and 6 and represents the “Normal” range of the input variable NGAL-U. The NGAL-U “Gray” membership function is triangular and is defined by the parameters [2, 50.0771, 70]. This triangular membership function peaks at approximately 50.0771 and tapers off to zero around 2 and 70 and represents the “Gray” range of the input variable NGAL-U. The NGAL-U “Path” membership function is trapezoidal and is defined by the parameters [10, 81, 100, and 106].

This trapezoidal membership function starts rising at 10, reaches full membership at 81, maintains full membership up to 100, and tapers off to 0 at 106 and represents the “Path” range of the input variable NGAL-U.

Figure 7 shows the membership function (MF) input variable Creat in MATLAB’s Fuzzy Logic Designer. The range of the input variable is from 0 to 4. The names of the membership functions are “Normal”, “Gray”, and “Path”. The Creat “Normal” membership function is triangular and is defined by the parameters [0, 0.5, and 1]. This triangular membership function peaks at 0.5 and tapers off to zero at 0 and 1 and represents the “Normal” range of the input variable Creat. Creat “Gray” membership function is triangular and is defined by the parameters [0.2, 0.8, 1.2]. This triangular membership function peaks at 0.8 and tapers off to zero at 0.2 and 1.2 and represents the “Gray” range of the input variable Creat. Creat “Path” membership function is trapezoidal and is defined by the parameters [0.6, 3, 4, 4]. This trapezoidal membership function starts rising at 0.6, reaches full membership at 3, maintains full membership up to 4, and tapers off to 0 at 4, and represents the “Path” range of the input variable Creat.

Figure 8 shows the membership function (MF) output variable Prob in MATLAB’s Fuzzy Logic Designer. The range of the output variable is from 0 to 1. The names of the membership functions are “Stationary”, “Gray”, “Progression”. The Prob “Stationary” membership function is triangular and is defined by the parameters [0, 0.15, and 0.3]. This triangular membership function peaks at 0.15 and tapers off to zero at 0 and 0.3 and represents the “Stationary” range of the output variable Prob.

The Prob “Gray” membership function is triangular and is defined by the parameters [0.3, 0.5, and 0.8]. This triangular membership function peaks at 0.15 and tapers off to zero at 0 and 0.3 and represents the “Stationary” range of the output variable Prob. The Prob “Progression” membership function is triangular and is defined by the parameters [0.8, 0.9, and 1]. This triangular membership function peaks at 0.9 and tapers off to zero at 0.8 and 1 and represents the “Progression” range of the output variable Prob.

### 2.3. Rule Base Creation

A rule base is essentially a collection of fuzzy IF–THEN rules that define the relationship between input variables and output variables.

A rule in fuzzy logic is an element that defines how the system should respond to different inputs. By using linguistic terms and combining conditions with logical operators, fuzzy rules provide a flexible and intuitive way to model complex decision-making processes that resemble human reasoning. A simple rule involves a single condition in the antecedent. A compound rule involves multiple conditions in the antecedent, connected by logical operators such as AND or OR. It is possible to assign assign weights to each rule to indicate their importance. Rule weights range from 0 to 1. This allows for fine-tuning the influence of each rule in the decision-making process.

A total of 94 rules have been written. At the end of each rule, a weight is attached, representing the importance that is given to each rule. An extract of rules configured in fuzzy system is presented in Table 4.

### 2.4. Inference Mechanism

The inference mechanism in a fuzzy logic approach is the process that applies the fuzzy rules to the input data to derive an output.

**Figure 9:**
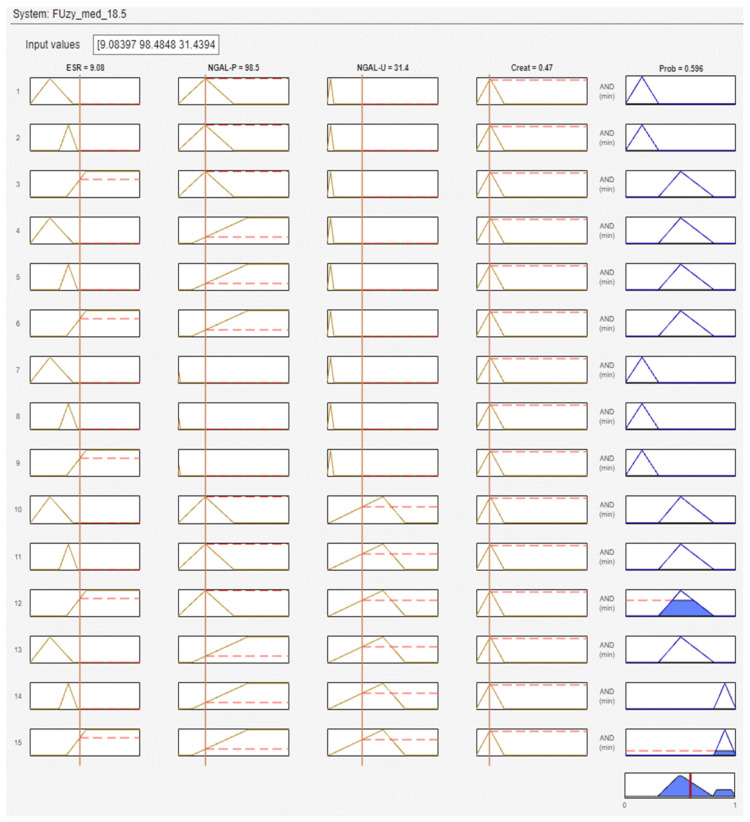
The Rule Inference tab (a screenshot of only 15 rules).

Figure 9 shows the Rule Inference tab in MATLAB’s Fuzzy Logic Designer. This tab provides a detailed view of how the fuzzy inference system (FIS) processes input values through its rules to generate output values. Each row represents the evaluation of a specific rule in the FIS. The columns show the degree of membership of each input variable in the respective membership functions (indicated by vertical lines on the plots). The resulting membership function for the output variable is shown in the last column, with the aggregated output.

Our proposed model is tuned for its current stage. Tuning a model involves adjusting the parameters of the fuzzy inference system to improve its performance, accuracy, or to better meet specific design requirements. The key aspects we covered for tuning the model were as follows:

*Membership Functions:* modifying the shape of membership functions and adjusting the parameters that define the membership functions.

*Rule Base:* changing the weights of individual rules to emphasize or de-emphasize their influence on the output and adding, removing, or altering rules to better capture the relationships between input and output variables. During the fuzzy inference process, the output of each rule is multiplied by its weight. This weighted output is then aggregated with the outputs of other rules to form the final fuzzy output.

*Inference Method:* choosing between different inference methods such as (Mamdani or Sugeno) to suit the problem’s requirements and modifying how the outputs of different rules are combined, e.g., max–min, sum–product.

*Defuzzification Method:* choosing the appropriate defuzzification method, such as centroid, bisector, mean of maxima, etc., to convert the fuzzy output back to a crisp value.

This stage of the model satisfies the needs of interpretation and discussion of the current results of our research.

Validating the tuned model with a separate validation dataset to ensure it performs well under various conditions will constitute a future approach as we accumulate an adequate volume of data.

## 3. Results

As can be seen in Figure 1, defuzzification is the final step in a fuzzy inference system where the fuzzy outputs are converted into a precise, crisp value. In the context of fuzzy logic and fuzzy inference systems, a “crisp output” refers to a precise, specific numerical value that is produced by the system as its final result. The centroid method is used for defuzzification. Most of the time, crisp output is presented graphically in the form of control surfaces. A control surface is a graphical representation of the output of a fuzzy inference system (FIS) with respect to its inputs. It is essentially a 3D surface plot that shows how the output varies as a function of two inputs, providing a visual way to understand and analyze the behavior of the fuzzy system. The control surface typically involves two input variables and one output variable. The input variables are plotted on the x and y axes, while the output variable is plotted on the z axis. Peaks, valleys, and slopes on the surface indicate regions where the output is significantly influenced by the inputs. This can help us understand the system’s sensitivity and performance.

Next, the results obtained by defuzzification are presented, regarding the correlations between the input and output variables. Each result is accompanied by an interpretation.

### 3.1. Correlation (Control Surface) ESR–Creat–Prob

**Figure 10:**
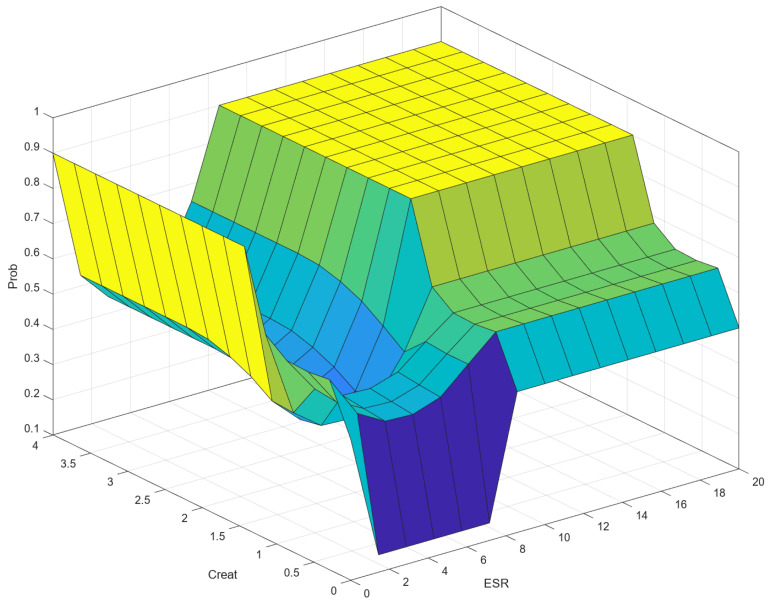
Correlation (Control Surface) ESR–Creat–Prob.

Figure 10 shows a 3D visualization of the relationship between the input variables ESR and Creat and the output variable Prob in the fuzzy inference system (FIS). The shape of the surface provides insights into the interaction between the input variables (regions where the surface is flat indicate areas where changes in input values have little effect on the output). Areas where the surface has steep gradients indicate that small changes in input variables lead to significant changes in the output variable. The following can be seen in Figure 10: as ESR increases, the value of Prob initially increases and then stabilizes for higher values of ESR; for lower values of Creat, Prob remains low regardless of ESR. As Creat increases, the effect of ESR on Prob becomes more pronounced. The surface suggests that there is a critical region where both ESR and Creat significantly influence the output Prob. The influence of ESR on Prob is more significant when Creat is above a certain threshold. This type of visualization helps us understand the behavior of the fuzzy inference system and identify critical regions where input variables have significant effects on the output.

### 3.2. Correlation (Control Surface) NGAL-U–Creat–Prob

**Figure 11:**
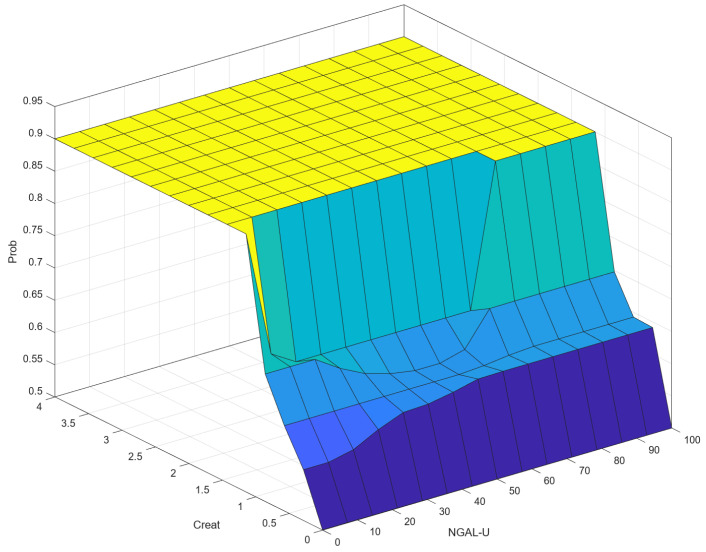
Correlation (control surface) NGAL-U–Creat–Prob.

Figure 11 shows a 3D visualization of the relationship between the input variables NGAL-U and Creat and the output variable, Prob. The following can be seen in the figure: For Creat values around 1.5 and above, Prob remains high (close to 0.9), indicating that Creat has a significant influence on Prob. For low Creat values (below 1.5), Prob remains low, showing that NGAL-U has a minor effect on the output unless Creat is high. NGAL-U has a noticeable effect on Prob when Creat is at higher values. Creat has a significant influence on Prob across its range, affecting “Prob” more substantially than “NGAL-U”.

### 3.3. Correlation (Control Surface) NGAL-P–Creat–Prob

**Figure 12:**
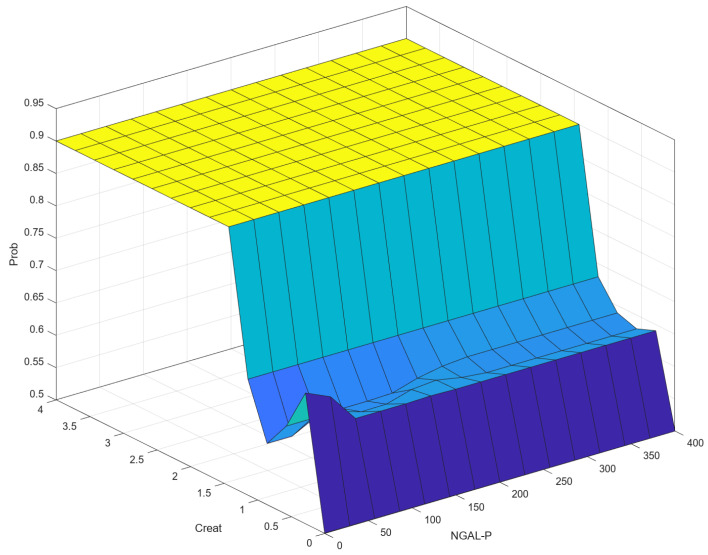
Correlation (control surface) NGAL-P–Creat–Prob.

Figure 12 shows a 3D visualization of the relationship between the input variables NGAL-P and Creat and the output variable, Prob. When the value of Creat is around 1.5 or above, Prob remains high (close to 0.9), indicating that Creat has a significant influence on Prob. For low Creat values (below 1.5), Prob remains low, showing that NGAL-P has a minor effect on the output unless Creat is high. 

### 3.4. Correlation (Control Surface) NGAL-P–NGAL-U–Prob

**Figure 13:**
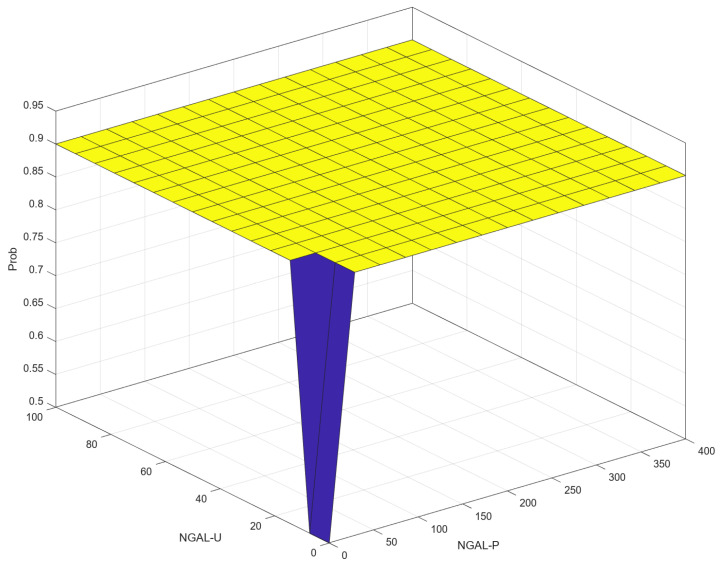
Correlation (control surface) NGAL-P–NGAL-U–Prob.

Figure 13 shows a 3D visualization of the relationship between the input variables NGAL-P and NGAL-U and the output variable, Prob. For both low values of NGAL-P and NGAL-U, the value of Prob is relatively low. When NGAL-P or NGAL-U increase, the value of Prob remains high: around 0.9. The plot shows that **except** for the very low values of both NGAL-P and NGAL-U, the Prob value is generally high (around 0.9). This may suggest that both variables have a combined effect on the output, with the lowest Prob observed when both inputs are at their minimum.

### 3.5. Correlation (Control Surface) ESR–NGAL-U–Prob

**Figure 14:**
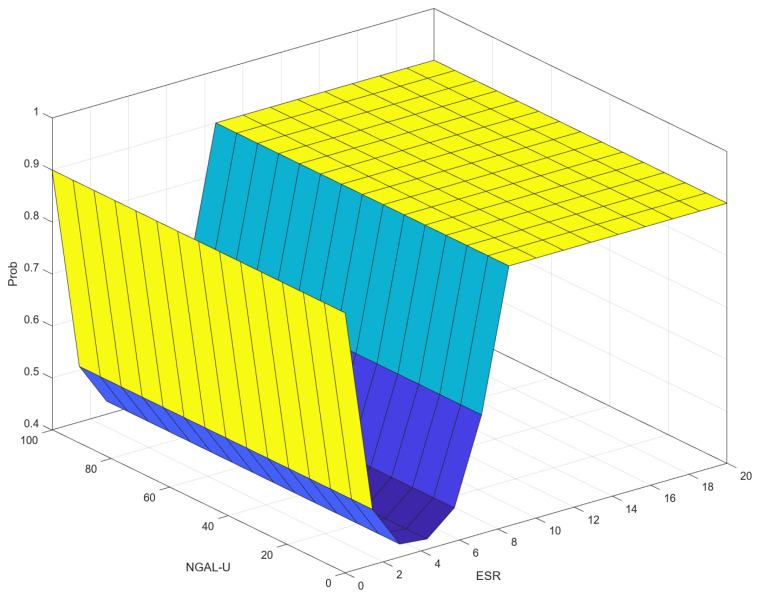
Correlation (control surface) ESR–NGAL-U–Prob.

Figure 14 shows a 3D visualization of the relationship between the input variables ESR and NGAL-U and the output variable, Prob. The highest values of Prob (around 0.9 to 1.0) occur when ESR is high, indicating a significant influence of ESR on Prob. When ESR is low, NGAL-U has a more pronounced effect on Prob, but as ESR increases, the impact of NGAL-U diminishes, and Prob remains high. NGAL-U has a noticeable effect on Prob when ESR is at lower values.

ESR has a significant influence on Prob across its range, particularly affecting Prob more substantially than NGAL-U as its value increases.

### 3.6. Correlation (Control Surface) ESR–NGAL-P–Prob

**Figure 15:**
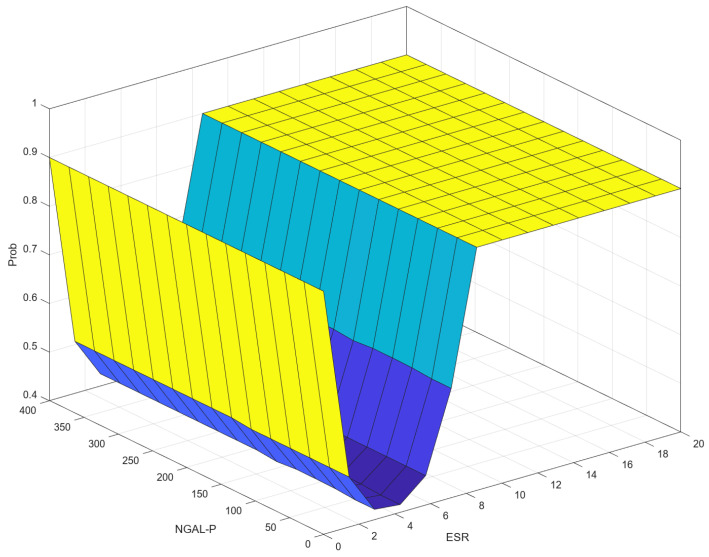
Correlation (control surface) ESR–NGAL-P–Prob.

Figure 15 shows a 3D visualization of the relationship between the input variables ESR and NGAL-P and the output variable, Prob. The highest values of Prob (around 0.9 to 1.0) occur when ESR is high, indicating a significant influence of ESR on Prob. When ESR is low, NGAL-P has a more pronounced effect on Prob, but as ESR increases, the impact of NGAL-P diminishes and Prob remains high. It seems that the correlation (control surface) for ESR–NGAL P–Prob and the correlation (control surface) for ESR–NGAL-U–Prob are similar.

## 4. Discussion

Fuzzy logic can be a very important tool in medicine and especially in diagnostics. This is because fuzzy logic is a soft computing technique which tolerates suboptimality and uncertainty, therefore producing very good results compared to other computational systems [20]. Fuzzy logic was first described by Lofti Zadeh in 1968 as a more adaptable alternative to Boolean logic [21]. Essentially, in fuzzy logic, statements are no longer absolute, i.e., true or false; rather, they are transitional values, with various degrees of truth associated with them. This is very similar to the diagnostic process, in which, at every level, differential diagnoses are possible [22].

In our multimodal modulation, we chose four parameters: serum creatinine (creat), urinary NGAL (NGAL-U), plasma NGAL (NGAL-P) and erythrocyte sedimentation rate (ESR). The reasoning for choosing these parameters will be discussed below. 

### 4.1. Serum Creatinine

At the time of our study, the traditional markers used for diagnosis and monitoring of chronic kidney disease, such as serum creatinine, eGFR, urea, and proteinuria, have important sensitivity limitations [2]. Theoretically, measuring GFR using inulin clearance is still the gold standard, but because this method is far too laborious, it is not routinely used in clinical practice. While GFR should reflect the number of functioning nephrons, there is evidence that the error calculated from serum creatinine varies from ±20% in adults and ±30–40% in children [36,37]. Furthermore, for patients aged below 2 years, eGFR is also underestimated due to the kidney’s immaturity [37]. This adds to the limitations of measuring endogenous creatinine represented by muscle mass, diet, and metabolism modifications (some of which may be induced by CKD itself) [2]. On the other hand, while NGAL has proven to be an independent marker of CKD in some studies, especially for the early stages [24], other authors consider that its values may vary depending on factors such as urinary output, water intake, or even the time of sample collection [15]. Accordingly, biomarker levels adjusted for creatinine may be more reliable [18]. Whilst some bias is related to monitoring solely serum creatinine, its value was chosen as a parameter for our fuzzy model instead of creatinine clearance due to the multitude of formulas, which would create more confusion/biases. It is expected that each laboratory/clinic will use their own approach to calculate creatinine clearance. The value of creatinine, however, should be standardized in hospitals over our country’s territory. Furthermore, this parameter can be changed in future research to reflect the local experience. 

One of our results regarding modeling for the progression of CKD using creatinine is shown in Figure 10. Here, the inflexion viewed for ESR values between 0 and 1 is due to the fact that it represents an absolute lack of inflammation. For this ESR interval, high creatinine values above 2 suggest progression, while gray creatinine values of 0.2–0.8 correlate for gray progression and creatinine values below 0.2 suggest no progression. The area between 0–0.5 Creat and 0.2–8 ESR suggests no progression. As ESR increases, in this interval, the progression probability rises to 0.4, remaining gray, but advising to the observer (medical practitioner) that patients whose results fall within these values should be further investigated. 

For ESR 2–8 mL/1 h and Creat 0.5–1.5, there are some areas of inflexion, maintaining progression in the gray area. As discussed, when defining the membership function areas for ESR, there is a gray area for ESR that overlaps with the range of normal values in which progression was observed. This is why the triangular membership function was chosen. As for the control surface area (Figure 10), we can see that this relationship of gray probability for ESR interval 2–8 is maintained for Creat values above 1.5 as well. The closer we come to the upper margin of the ESR interval, the more the probability of progression increases. Finally, for Creat values above 2, with ESR values above 12, there is a critical region of progression of CKD, meaning that when creatinine and ESR values are high there is progression of CKD, which is supported by the literature [38].

### 4.2. Neutrophil Gelatinase-Associated Lipocalin (NGAL-P and NGAL-U)

NGAL is a small lipoprotein (25–135 kDa) belonging to the lipocalin family. There are three known forms of NGAL: the kidney 25 kDa monomer, the neutrophil 45kDa heterodimer, and the 135 kDa metalloproteinase complex [39,40,41]. It can be present both in serum and in urine [19]. NGAL was initially identified in neutrophils, but also has significant expression in the kidney, liver, heart, stomach, adipose tissue, and epithelium [19]. Thus, increased NGAL levels will be found alongside inflammation, infections, poisoning, ischemia, kidney cell damage and neoplasia [19]. Both plasma and urinary fractions have been associated with various medical conditions such as sepsis [42], various cancers [43,44,45], coronary disease [46], arterial hypertension [47], diabetes [48], and also acute kidney injury as a response to tubular injury and are involved in the renal regeneration processes [9,19]. 

The 25 kDa monomer is produced by renal tubular cells [49]. At the moment of kidney injury, NGAL secretion will be significantly increased in the distal tube (Henle loop, distal tube, collector tube), thus promoting an increase in urinary NGAL (uNGAL). Its expression is also increased in the regenerating tubular epithelial cells. uNGAL can also mean proximal tubular cell destruction [25]. The plasmatic NGAL (pNGAL) increase has multiple sources from the inflammatory process linked to the kidney injury to secretion (neutrophils, macrophages) but also through resorption in the kidney tubular system [4,19].

The progression of chronic kidney disease is marked by progressive glomerular sclerosis, tubulointerstitial fibrosis, and progressive tubular lesions and thus provides increases in both fractions [4,19].

Regarding the progression of chronic renal pathology, a study conducted in an adult population concluded that both serum and urinary NGAL monitoring can be considered as a strong independent predictor of progression and after adjustments for eGFR [24]. The study was conducted on a group of 96 adult patients with periodic re-evaluations lasting 18.5 months. NGAL was a predictor of progression of chronic kidney disease and showed the severity of kidney disease at earlier stages. This study has important limitations. Although patients with progression have elevated NGAL levels, due to the small number of patients, it is not possible to assess the level of impact of the pathological substrate (primary diseases) on the progression of chronic kidney disease and a correlation with NGAL level. At the same time, the patients introduced in the study did not represent a typical distribution of a population with chronic kidney disease, and the reassessment time was relatively short [24].

Other authors consider the utility of the uNGAL/creatinine ratio superior to uNGAL alone in progression monitoring, as it improves the prediction of the progression of kidney disease [18,50] in these patients, and NGAL plasma levels also predict the progression of chronic kidney disease at later stages (3 and 4). Smith ER et al. observed that urinary NGAL levels and a uNGAL/creatinine ratio were associated with higher risk of death and initiation of extrarenal replacement therapy independent of renal and cardiovascular risk factors in 158 adult patients with stage 3 or 4 chronic kidney disease [50].

In small studies, NGAL alone was a high-performance biomarker. Mitsnefes MM et al. observed an inversely proportional association between plasma NGAL levels and the estimated glomerular filtration rate in 45 pediatric patients with stage 2–4 chronic kidney disease. For a glomerular filtration rate of less than 30 mL/min, NGAL was even more effective than cystatin C in monitoring kidney function [51]. 

Urinary NGAL has shown promise in monitoring CKD progression in patients with urinary malformations [52,53]. It has also shown utility in monitoring CKD progression in patients with glomerular etiology, such as nephrotic syndrome, where it can also be used to differentiate between minimal change nephrotic syndrome and focal and segmental glomerulosclerosis and treatment toxicity [28,54,55]. uNGAL is also monitored in CKD progression determined by IgA nephropathy [34] and systemic lupus erythematosus [56], while plasma NGAL is used to detect CKD progression in diabetic nephropathy [35].

Regardless of their origin and associated risk factors, pathophysiological phenomena converge in the direction of a common tubulo-interstitial involvement characterized by tubular atrophy and hypoxia, peritubular capillary involvement, and interstitial fibrosis [2,4]. Given the crucial role played by the renal tubular apparatus described above, the following question arises: could NGAL dosing be more effective (sensitive) when evaluating patients with chronic renal pathology? And if so, this could be continued by the following questions: In which of all the etiologies of chronic kidney disease could dosing be more effective? It shows promise both in certain cases where the underlying pathology is represented by hereditary renal disorders such as vesicoureteral reflux and congenital obstructive uropathy manifested by uretero-hydronephrosis [57,58] and in pathologies of glomerular origin [56]. The advantages of knowing about the behavior of NGAL in this population are manifold. On the one hand, it allows therapy to be adjusted in order to slow down the progression of the disease. At the same time, it could be used in the early determination of intensifying re-evaluation intervals in order to intervene early. Last but not least, there is the possibility of establishing the need for imaging (scintigraphy) or biopsy re-evaluations depending on a possible interpretation of plasma values compared to urinary values of NGAL or to other known tests such as ESR or creatinine.

Both urinary and plasma fractions of NGAL have been proven to increase in both AKI and CKD. Their cutoff points and specificity and sensitivity levels have yet to be agreed upon. They both show promises in diagnosis [24], but also for assessing disease activity [27,37], predicting outcomes [26,59,60], and monitoring treatment response [28] in kidney diseases, regardless of etiology. Further research is needed to validate its clinical utility and establish standardized thresholds for interpretation in this patient population. 

NGAL does not have a universally agreed-upon set cutoff point specifically for CKD. The interpretation of NGAL levels in CKD is complex and requires consideration of various factors, including the underlying etiology of kidney disease, the stage of CKD, the presence of comorbidities, and the clinical context of the individual patient. It is essential to interpret NGAL levels in conjunction with clinical and laboratory findings, including serum creatinine and imaging studies, to obtain a comprehensive assessment of kidney function and disease severity. An example of this is illustrated in studies by Anand et al. [61] that provided a median value of uNGAL of 281.2 ng/dL for CKD progression, while Eskandarifar et al. [62] suggested values of 524.05 ± 166.65 ng/dL for CKD progression. Both studies had patients with congenital anomalies of kidney and urinary tract (CAKUT) as underlying etiology of CKD. This lack of consensus illustrates the limitations of the statistical approach.

Observing the large utility of NGAL in various etiologies of CKD and the varied results in the literature, we have tried to mimic the patient distribution to include as varied etiologies as possible. We considered this in order to obtain as many and as different results for value intervals for our biomarkers as possible, and as such to mirror the reality of the clinician. Considering different modifications in CKD, we consider it wise to monitor both seric and urinary modifications. Patients were admitted into the study with maximum limitations of confounding sources (e.g., no infections at the moment of sample collection, no other source of inflammation other than CKD). Etiology in itself provided some modifications e.g., Nephrotic syndrome promotes an increase in leucocytes in urine, which, consequentially, will promote an increase in uNGAL values. 

We believe our multimodal design mimics clinical reality. In our multimodal design, based on our data (Figure 11) we observe that for a creatinine value between 0.5 and 1.5, variations in the correspondent value of NGAL can link the probability of progression from low to medium and close to high, suggesting that the model is consistent with the current literature and can provide further clinical aid.

In Figure 10, we must note that for Creat values of 0–0.2, any variation in NGAL-U does not suggest the probability of CKD. This is because while NGAL-U is mainly secreted in tubular injury, some situations may occur in which NGAL-P is overly secreted, passes through the glomerular filter, and is insufficiently resorbed [4,19,25].

The more Creat rises and NGAL-U rises, the more the probability of CKD increases. This is in consensus with Bolignano et al. 2009 [24], who found a correlation between uNGAL and CKD progression, especially at lower stages. This correlation is further illustrated for the interval of Creat (0.5–1.5) where, whilst the progression risk still increases, if NGAL-U is situated in the gray membership function interval, the probability of progression diminishes. This is also in consensus with Bolignano et al. [24]. 

There is consensus that there is a relationship between NGAL-P and NGAL-U, though the precise mechanisms behind it are unknown [9,10,19,24]. This is also illustrated in Figure 12 compared to Figure 10, and furthermore in Figure 13.

Figure 12 shows the influence of probability by correlation of NGAL-P and creatinine. For Creat at an interval of 0–0.2, variation of NGAL-P does not suggest progression. As NGAL-P can be secreted by multiple sources, variation of NGAL-P can be present in the absence of renal involvement (damage). For Creat 0–0.5, we can see an initial inflexion for the probability for NGAL-P that is situated in the 0–50 interval. This is because in this area it is considered that no sufficient renal modification suggests that NGAL-P is of renal origin. As NGAL-P values increase, correspondent to the creatinine interval, we can see that we move to a gray area of progression. This is in concordance with the stipulation that NGAL-P values increase prior to rises in creatinine levels [9,10,19,24]. As we move closer to higher Creat levels, we can see that probability of CKD increases with the increase in NGAL-P values. This is also in concordance with the findings of Bolignano et al., 2009 [24].

Figure 13 shows the influence of NGAL-P and NGAL-U values on progression. It shows that for a patient that has NGAL-P and NGAL-U values in the gray area, there is a very high risk of progression. We consider this distribution as valid, based the available literature [9,10,24,25]. The whole premise is that when starting with a certain value of one or another, the risk of progression increases. While we consider this figure to support the others, a few limitations must be considered. First of all, we consider this result to be mainly influenced by the design of our rules, in which we suggest more relevant expression of renal damage if NGAL-U values are outside the intervals which we consider normal in the membership functions over the risk of progression. This is our interpretation of the literature that suggests that increases in urinary NGAL are less likely to be influenced by external factors than increases in plasma NGAL [19]. The figure, however, does illustrate the aforementioned demonstrated correlation between plasma and urinary NGAL values [9,10,19,24]. We must also take into consideration that, in clinical practice, doctors may not have both NGAL-U and NGAL-P available at the same time, so it may be of limited specific use.

### 4.3. Erythrocyte Sedimentation Rate (ESR)

Finally, CKD progression varies with underlying etiology. For malformations, current diagnostic and monitoring emphasis lies in imagistic investigations. Both glomerular and tubular etiologies cause chronic inflammation, which, in turn, will stimulate secretion of NGAL [19]. It is important to differentiate baseline NGAL values and monitor modifications in accordance with these values. Erythrocyte sedimentation rate (ESR) or ferritin should be monitored in parallel to potentially evocate inflammatory status modifications.

CKD is a local disease with systemic impact, including triggering of inflammatory response. As such, we need a parameter to mirror its impact. There are numerous biomarkers that are specific to some points of the inflammation cascade. However, they are not routinely used or universally available. This, in itself, contradicts one of the main objectives of the study. Our aim was to develop a simple diagnostic aid, using fairly routine blood and urine tests, in conjunction with our biomarkers, and thus enabling accessibility. Erythrocyte sedimentation rate (ESR) is sensitive, whilst highly non-specific, but in its usage we can evaluate the involvement of the inflammatory link from CKD pathophysiology [38]. In conjunction with NGAL physiopathology, it creates a system that allows for explanation of value modifications of non-renal causes.

Our multimodal approach for CKD progression based on ESR and creatinine value also seems to mimic the clinical reality. For a creatinine superior to 1.5 with an ESR level considered normal (2–8), we obtained a gray area of progression (Figure 10). We interpret that area to be likely attributed to the precise acute kidney injury moment which, in theory, is a different event that causes inflammation in the aftermath. In clinical practice, in that particular situation, it is unlikely that inflammation does not occur. On the other hand, for increased inflammation (ESR > 8) and creatinine in the aforementioned interval, our model is linked to CKD progression. Furthermore, increased inflammation with no creatinine modification is situated in the gray area, as you can neither link it 100% to CKD progression nor deny its implication.

Figure 14 and Figure 15 show the impact on progression by ESR and either NGAL-U or NGAL-P. As discussed earlier, in clinical practice, one does not obtain an ESR of zero. However, in Figure 14, the model shows that in the complete absence of inflammation, the progression probability rises with NGAL-U, as it is most certainly of renal origin. The inflexion is natural and shows a very low probability of renal involvement at normal ESR intervals, which increases as ESR reaches gray membership. As ESR increases, and NGAL-U values increase, so does the probability of CKD. The same is to be said for Figure 15 distribution regarding NGAL-P, further illustrating the relationship between NGAL-U and NGAL-P that has been demonstrated in the literature [9,10,19,24]. The correlation with ESR is to be normal, as ESR is a marker for inflammation, which is a key process both in NGAL secretion and in CKD progression [9,10,19].

### 4.4. Fuzzy Logic and the Multimodal Approach

We should note that all the parameters described above both have advantages and limitations and that having them work in conjunction will determine a far more accurate assessment of the patients’ biological status. This is where the fuzzy logic shines.

The concept of fuzzy logic has been adapted in medicine before [63], but never for pediatric patients, nor for novelty biomarkers.

Fuzzy logic in medicine allows for the usage of nuances in the decision process instead of the inflexible “yes” and “no”. The final result of the process is a map/diagnostic model. Fuzzy logic has been used in medicine before in respiratory diseases (asthma, COPD), metabolic diseases (diabetes mellitus), cardiovascular, or gynecological diseases [23].

Ahmed Abou Elfetouh Saleh et al., 2011 proposed a fuzzy decisional support system for identifying and providing a risk assessment of breast cancer. They used variables such as the presence of hormonal receptors, human epidermal growth factor receptor 2 values, age, tumor grade, and lymph nodes to determine risk [64].

Badnjevic A et al., 2015 used fuzzy logic for classification of asthma and COPD patients’ Impulse Oscillometry System based on test results and spirometry and achieved a more accurate classification [65].

Finally, more recently, Maad Shatnawi et al., 2021 proposed an intelligent fuzzy inference system for the primary diagnosis of COVID-19, based on patient symptoms [66].

While this approach is not new, in medicine, it has not been used for pediatric CKD progression monitoring. 

We propose this model for the clinician, who should use the entirety of control surface areas in order to improve diagnosis and choices regarding follow-up and, eventually, therapy. 

The model has numerous advantages. Firstly, it allows for a common ground regarding CKD progression evaluation using these two biomarkers. Secondly it improves the accuracy of perspective regarding CKD progression states. Lastly, it provides further support to doctors for the need of personalized follow-up and reduced re-evaluation intervals.

There are certain limitations of this study. Considering this is a novel approach in literature there were no guiding landmarks regarding parameters. This is the first integrative approach when considering the blood parameters. The exact correlation and corresponding weight between them can at this point solely be hypothesized. Also, as is the case for all fuzzy logic models, there is the issue regarding the “black box” of the rules, the subjectivity in formulation those rules, and generating the cut-off point [20,22]. The latter two inconveniences can be managed by hybridization of the system with a neural network, a task that is unwarranted at this time because of the limited number of cases. The population we tested is small, but with relatively different CKD etiologies and relatively close CKD stages. CKD progression was defined as a greater than 4 mL/min/1.73 m^2^ decrease in eGFR, but this did not necessarily indicate progression into the latter stages of CKD. Given the non-linear degree of CKD progression, we recommend further testing for validation in more varied populations. Also, while we did define it, we would prefer if the clinician would also interpret the term of CKD progression as “proof of ongoing lesion that needs further/closer investigation”. The current literature has both sNGAL and pNGAL studies values. Studies [17,67] suggest there is a clear and strong correlation between the two fractions, but that value ranges may not be equivalent. For the purpose of our study, we chose to validate with pNGAL, which is considered to have lower values [17], and as such makes for more sensitive predictions. Validation may need to consider using sNGAL as well.

Lastly, membership functions are relative to our patient values and current available literature regarding NGAL in CKD. As knowledge increases, the values and rules may need updating. 

Considering our patient data and correlations with medical literature, we believe we have successfully developed a prototype of a valuable future diagnostic tool. 

## 5. Conclusions

Current monitorization methods for pediatric CKD are limited. New biomarkers show some promise, but values are not standardized for children. CKD case incidence and progression are increasing, as suggested by the increased number of renal replacement therapy patients of pediatric age. The need for further interpretation of the new biomarker available data is evident. Our study provides a potential solution, by a novel approach both by using these particular biomarkers and as a novel way of using fuzzy logic as a diagnostic tool in pediatric CKD.

Our study took into consideration available literature and personal data regarding uNGAL and plasma/serum NGAL and the impact of routinely used blood parameters (creatinine and ESR) on the progression of CKD and proposed a more clinical, non-limiting interpretation of their impact on CKD progression. We identified that at this point in time, there is no consensus in the literature regarding the values of these biomarkers and that the statistical approach is limited. As such, we proposed a fuzzy logic model to aid the clinician. 

In our study, we described in detail how to configure a fuzzy model that can simulate the correlations between the input variables ESR, NGAL-P, NGAL-U, creatinine, and the output variable Prob regarding the prognosis of the patient’s evolution.

The results of the simulations on the model, i.e., the correlations between the input and output variables (3D graphic presentations), were explained in detail.

All the correlations between the input and output variables were commented/analyzed by referring to the own experimental data and those from the identified specialized literature.

To the best of our knowledge, this is the first model of CKD progression monitoring using these biomarkers in the context of fuzzy logic, and we believe it can be of great value for clinicians in improving diagnosis, patient monitoring, and choices regarding follow-up and eventually therapy. Physicians can base the situation of the patient on the scales we proposed. If the patient is in a gray area (light blue), we propose reducing follow-up intervals. In case of progression (yellow), other measures to reduce further progression, relative to the stage of CKD, can be taken into consideration.

A fuzzy approach should provide further insight in specific situations when patients may need modifications in either follow-up intervals or therapeutic changes and, as such, alleviate the financial burden and improve the quality of life of these patients.

Further research can be continued by expanding to the neuro-fuzzy approach, which enables trainable diagnostic models.

## Figures and Tables

**Table 1:** Patient value distribution.

	Average	Median	Range
Age (years)	7.3	6	2–16
eGFR (mL/min/1.73^2^)	119.01	112.5	14.77–251.42
Serum creatinine (mg/dL)	1.3	0.56	0.21–7.77
pNGAL (ng/mL)	647.66	484.14	204.69–1415
uNGAL (ng/mL)	52.7	46.84	10.38–97.96
ESR (mL/2 h)	14.29	6	2.00–91

**Table 2:** Patient characteristics regarding fuzzy logic parameters and associated rules.

Patient	ESR (mm/2 h)	NGAL-P (ng/mL)	NGAL-U (ng/mL)	Creat (mg/dL)	Progression	Rule
1	12	943.7302	88.27665	0.81	Prog	78
2	91	854.1498	97.96497	0.55	Prog	51
3	2	340.03662	did not give sample	0.21	Gray	4
4	5	204.6919	79.70809	0.56	Prog	47
5	21	388.7212	13.73992	0.5	Prog	42
6	6	818.1229	20.84793	1.25	Prog	68
7	12	1415.001	88.95824	7.77	Prog	78
8	25	595.1456	24.88878	0.44	Prog	42
9	5	484.1438	10.38065	0.55	Prog	68
10	11	423.7744	76.68962	0.26	Prog	24
11	6	362.4313	19.33869	0.52	Prog	41
12	14	1261.1564	93.43726	4.47	Prog	78
13	5	541.5921	35.74554	0.96	Prog	41
14	6	304.0093	52.49318	0.61	Gray	38
15	11	1266.0249	81.02259	1.47	Prog	77
16	5	356.5891	18.60842	0.66	Prog	41
17	6	451.038	41.19826	0.54	Prog	50

**Table 3:** Reported values of NGAL in literature.

Study	pNGAL/sNGAL (ng/mL)		uNGAL (ng/mL)	
	Normal	Pathological	Normal	Pathological
Bolignano et al., 2009 [24]	18.9–46.5	58.9–1405.5	2.1–9.6	4.1–801.6
Bolignano et al., 2008 [25]	-	-	4.54–10.64	267.15–489.48
Rafiei A. et al., 2015 [26]	-	-	-	7.32
Kim B.K. et al., 2017 [27]	-	261.1–418.2	-	-
Gacka E et al., 2016 [28]	-	-	-	5–72
Ozkan S et al., 2014 [29]	-	134–722	-	-
Tuan PNH et al., 2020 [30]		273.73–613.74		138.92–734.59
Basturk T et al., 2017 [31]	-	35.95–278.99	-	-
Lindberg S et al., 2016 [32]	-	88–291	-	-
Seibert FS et al., 2021 [33]	-	-	11.97	14.86
Ding H et al., 2007 [34]	-	-	-	20–30
Bolignano et al., 2009 [35]	9.2–88.9	440–1340	1.8–22.3	6.2–768

**Table 4:** Example of rules configured in fuzzy system (an extract).

Rules	Weight of Rule
4 If ESR is Normal and NGAL-P is Path and NGAL-U is Normal and Creat is Normal then Prob is Gray	0.2
24 If ESR is Path and NGAL-P is Path and NGAL-U is Path and Creat is Normal then Prob is Progression	1
38 If ESR is Gray and NGAL-P is Gray and NGAL-U is Gray and Creat is Gray then Prob is Gray	1
41 If ESR is Gray and NGAL-P is Path and NGAL-U is Gray and Creat is Gray then Prob is Progression	0.8
42 If ESR is Path and NGAL-P is Path and NGAL-U is Gray and Creat is Gray then Prob is Progression	1
47 If ESR is Gray and NGAL-P is Gray and NGAL-U is Path and Creat is Gray then Prob is Progression	0.7
50 If ESR is Gray and NGAL-P is Path and NGAL-U is Path and Creat is Gray then Prob is Progression	0.8
51 If ESR is Path and NGAL-P is Path and NGAL-U is Path and Creat is Gray then Prob is Progression	1
68 If ESR is Gray and NGAL-P is Path and NGAL-U is Gray and Creat is Path then Prob is Progression	1
77 If ESR is Gray and NGAL-P is Path and NGAL-U is Path and Creat is Path then Prob is Progression	1
78 If ESR is Path and NGAL-P is Path and NGAL-U is Path and Creat is Path then Prob is Progression	1

## Data Availability

The simulation files/data used to support the findings of this study are available from the corresponding author upon request.
